# Vascular function and cardiovascular risk in a HIV infected and HIV free cohort of African ancestry: baseline profile, rationale and methods of the longitudinal EndoAfrica-NWU study

**DOI:** 10.1186/s12879-020-05173-6

**Published:** 2020-07-03

**Authors:** Carla M. T. Fourie, Shani Botha-Le Roux, Wayne Smith, Aletta E. Schutte, Yolandi Breet, Carina M. C. Mels, Lebo F. Gafane-Matemane, Leandi Lammertyn, Lisa Uys, Adele Burger, Jitcy S. Joseph, Nandu Goswami, Patrick De Boever, Hans Strijdom

**Affiliations:** 1 Hypertension in Africa Research Team (HART), North-West University, Private Bag X1290, Potchefstroom, South Africa; 2 South African Medical Research Council: Unit for Hypertension and Cardiovascular Disease, Faculty of Health Sciences, North-West University, Potchefstroom, South Africa; 3 School of Public Health and Community Medicine, University of New South Wales, The George Institute for Global Health, Sydney, Australia; 4 Toxicology and Biochemistry Department, National Institute for Occupational Health, Johannesburg, South Africa; 5 Gravitational Physiology and Medicine Research Unit, Division of Physiology, Medical University of Graz, Graz, Austria; 6 Health Unit, Flemish Institute for Technological Research (VITO), Mol, Belgium; 7 Centre for Environmental Sciences, Hasselt University, Diepenbeek, Belgium; 8 Department of Biology, University of Antwerp, Wilrijk, Belgium; 9 Centre for Cardiometabolic Research in Africa, Division of Medical Physiology, Faculty of Medicine and Health Sciences, Stellenbosch University, Stellenbosch, South Africa

**Keywords:** HIV, Antiretroviral therapy, Cardiovascular risk markers, Endothelial function, Vascular function, African ancestry, South Africa

## Abstract

**Background:**

People living with the Human Immunodeficiency Virus (PLHIV) have an increased susceptibility to develop non-communicable diseases such as cardiovascular disease (CVD). Infection with HIV contributes to the development of CVD independent of traditional risk factors, with endothelial dysfunction being the central physiological mechanism. While HIV-related mortality is declining due to antiretroviral treatment (ART), the number of deaths due to CVD is rising in South Africa - the country with the highest number of PLHIV and the world’s largest ART programme.

The EndoAfrica study was developed to determine whether HIV infection and ART are associated with cardiovascular risk markers and changes in vascular structure and function over 18 months in adults from different provinces of South Africa. This paper describes the rationale, methodology and baseline cohort profile of the EndoAfrica study conducted in the North West Province, South Africa.

**Methods:**

In this case-control study, conducted between August 2017 and June 2018, 382 volunteers of African descent (276 women; 106 men), comprising of 278 HIV infected and 104 HIV free individuals were included. We measured health behaviours, a detailed cardiovascular profile, and performed biomarker analyses. We compared baseline characteristics, blood pressure, vascular function and biochemical markers between those infected and HIV free.

**Results:**

At baseline, the HIV infected participants were older (43 vs 39 years), less were employed (21% vs 40%), less had a tertiary education (7% vs 16%) and their body mass index was lower (26 vs 29 kg/m^2^) than that of the HIV free participants. While the cardiovascular profile, flow-mediated dilation and pulse wave velocity did not differ, glycated haemoglobin was lower (*p* = 0.017) and total cholesterol, high density lipoprotein cholesterol, triglycerides, gamma-glutamyltransferase and tobacco use were higher (all *p* < 0.047) in PLHIV.

**Conclusion:**

Despite PLHIV being older, preliminary cross-sectional analysis suggests that PLHIV being treated with ART do not have poorer endothelial or vascular function compared to the HIV free participants. More detailed analyses on the baseline and follow-up data will provide further clarity regarding the cardiovascular profile of South Africans living with HIV.

## Background

The global fight against the acquired immunodeficiency syndrome (AIDS) epidemic has largely been successful with the number of human immunodeficiency virus (HIV) related deaths declining faster than the global rate of new infections. However, much work is still required [1]. The number of people living with HIV (PLHIV) has grown to 37,9 million, and 800,000 of the 1,7 million new infections in 2018 were in eastern and southern Africa, which is home to 54,4% of the world’s PLHIV [1]. South Africa had approximately 7,97 million PLHIV in 2019, which is the highest number globally, with an HIV prevalence rate of approximately 19% in adults (15–49 years) [2]. Consequently, South Africa also has the largest antiretroviral treatment (ART) programme in the world with 62% of the adult PLHIV receiving ART [1].

Apart from the high prevalence of PLHIV, the impact of cardiovascular disease (CVD) on the sub-Saharan African population [3] is placing additional strain on the public health care system. It is clear that ART has improved the morbidity and mortality associated with HIV, however, PLHIV are exposed to adverse cardiovascular events due to a complex interplay between HIV, ART and CVD [4]. Cardiovascular disease has become a common cause of death in PLHIV as the population grows older [5]. In South Africa, non-communicable disease death rates are higher than that of HIV [6, 7], with CVD being the most prominent.

One of the main risk factors for the development of CVD is vascular dysfunction. In PLHIV this is clearly evidenced by reports of impaired endothelial (reduced flow-mediated dilation (FMD)) [8], and vascular function (increased large artery stiffness and decreased distensibility) [9]. Furthermore, significant variations in the microvasculature, such as the retinal vascular beds [10], increased carotid wall thickness [11] and electrocardiogram (ECG) abnormalities, which were predictive of incident CVD [12], were reported in PLHIV.

Endothelial dysfunction is characterised by increased endothelial permeability, leading to increased inflammatory cytokine levels and adhesion molecule expression [13]. The mechanisms contributing to HIV-related endothelial and vascular dysfunction are multifaceted. This could include a combination of a HIV induced increase in reactive oxygen species and reduced nitric oxide bioavailability affecting endothelium-dependent vasodilatation, direct HIV infection of the endothelium, viral related endothelial activation, and vascular inflammatory effects due to systemic inflammatory cytokine or chemokine dysregulation [13–15]. Furthermore, recent studies indicate that the HIV viral protein, gp120, reduces nitric oxide, and the HIV Tat protein induces endothelial oxidative stress, activates apoptosis of both endothelial cells and cardiomyocytes, and upregulates the expression of cellular adhesion molecules [13].

It is well known that ART successfully lowers the viral load and decreases the mortality rate, however, it is unclear how ART affects endothelial and vascular function. Although treatment was associated with increased stiffness [9], Solages et al. demonstrated worse endothelial function among those with elevated levels of HIV replication [8]. Furthermore, most of the reported research was not done in African populations; we have shown in South Africa that HIV infection is related to increased endothelial activation [16]. The odds of having increased endothelial activation were more prominent in those without treatment than in their treated counterparts [16].

Our understanding of the complex interaction between HIV and ART in the progression of CVD has improved, however, more detailed exploration into the mechanisms of HIV-induced endothelial dysfunction is needed [13]. A better understanding of HIV-specific biomarkers may contribute to the development or improvement of guidelines, such as the European AIDS Clinical Society guidelines, to guide health care providers in their approach to prevent and treat CVD in PLHIV [13].

To enhance the knowledge base on HIV, ART, endothelial dysfunction and CVD in South Africa, Strijdom et al. commenced the parent EndoAfrica study titled “Vascular endothelial dysfunction: The putative interface of emerging cardiovascular risk factors affecting populations living with and without HIV in Sub-Saharan Africa” in April 2015 [17]. However, the parent EndoAfrica study executed in the Western Cape, South Africa, largely recruited patients of mixed ancestry; challenging the extrapolation of the findings to the broader South African population [17]. We expanded on the parent study by including participants of African ancestry from the North-West province. The North-West province leg of the EndoAfrica study, EndoAfrica-NWU study, thus now provides an opportunity to broaden the relevance of the findings to a larger proportion of the Sub-Sahara African population.

The EndoAfrica-NWU study aims to determine whether HIV and first-line ART regimens are associated with CVD risk factors and changes in cardiovascular structure and function in adults living in the North West province of South Africa. Analyses on macro vascular structure and micro vascular function and structure is ongoing. Therefore, in this study, we describe the rationale, methods and baseline characteristics, blood pressure, vascular function and biochemical markers of the EndoAfrica-NWU study.

## Methods

### Study design and setting

**Fig. 1:**
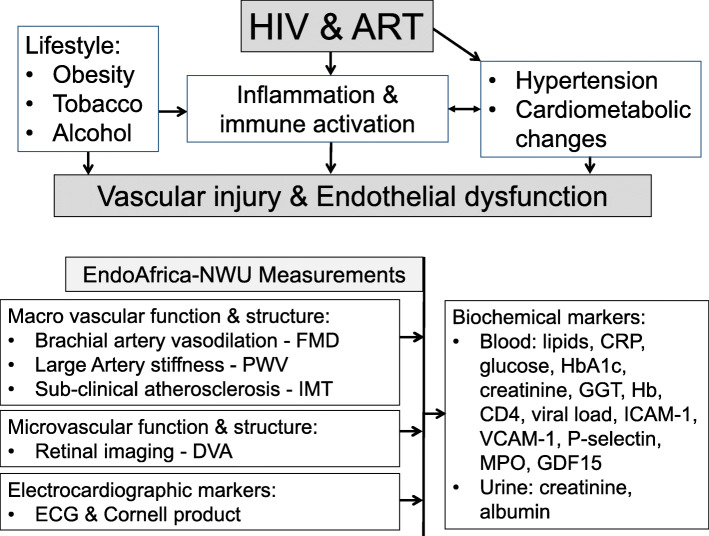
Rational for measurements and analyses in the EndoAfrica-NWU study. HIV: human immunodeficiency virus; ART: antiretroviral treatment; FMD: flow-mediated dilation; PWV: pulse wave velocity; IMT: intima-media thickness; DVA: dynamic vessel analysis; ECG: electrocardiography; CRP: C-reactive protein; HbA1c: glycated hemoglobin; GGT: γ-glutamyltransferase; Hb: hemoglobin; ICAM: intercellular adhesion molecule; VCAM: vascular cell adhesion molecule; MPO: myeloperoxidase; GDF-15: growth differentiation factor-15.

The design of the parent EndoAfrica study was discussed in detail by Strijdom et al. [17]. The North West province leg of the study (EndoAfrica-NWU) will adhere to the same design as the parent study, but within a population of African descent. The rationale for the study is summarised in Fig. 1. The study design and setting will be discussed in subsequent sections.

**Fig. 2:**
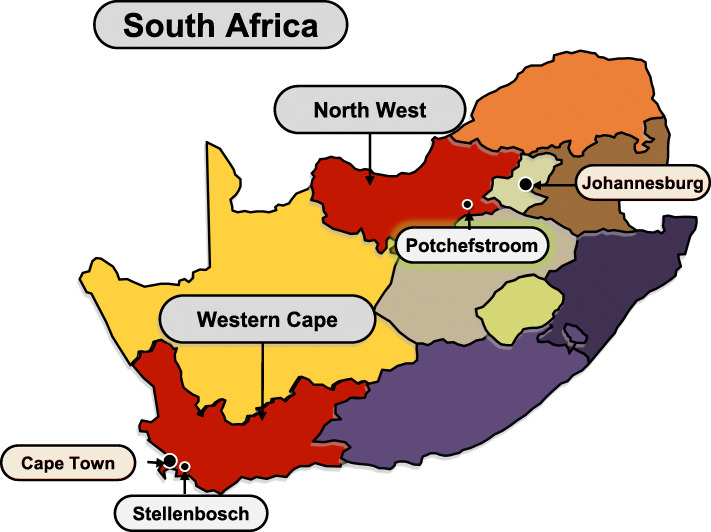
Data collection for the EndoAfrica-NWU study took place in Potchefstroom within the North West province, South Africa. Source of the editable map *Servier Medical Art* (https://smart.servier.com/)

For the EndoAfrica-NWU study, participants were recruited in and around Potchefstroom, North West province, South Africa (Fig. 2). In 2018, South Africa’s population was estimated at 57,73 million people of diverse origins and cultures. Of the total South African population, 80,9% declared themselves to be black Africans (African ancestry), 8,8% coloured (mixed ancestry), 7,8% white and 2,5% Indian or Asian [2]. While the mixed ancestry and white South Africans make up the majority of the population in the Stellenbosch (Western Cape) area where the parent EndoAfrica study was executed, the majority of people living in the Potchefstroom area is black South Africans (77,1% based on the 2016 census data).

### Participant recruitment and eligibility

The study was approved by the Health Research Ethics Committee of the North-West University (NWU) (ethics number: NWU-00045-15-A1) and the North West Department of Health. Goodwill permission to visit the clinics was granted by the North West Province of Health sub-district and the Potchefstroom Patient Group of the local hospital gave permission to recruit from the clinics. Participants were recruited at seven clinics in and around Potchefstroom and at HIV support groups by circulating leaflets (English and Setswana) as well as by word-of-mouth.

**Fig. 3:**
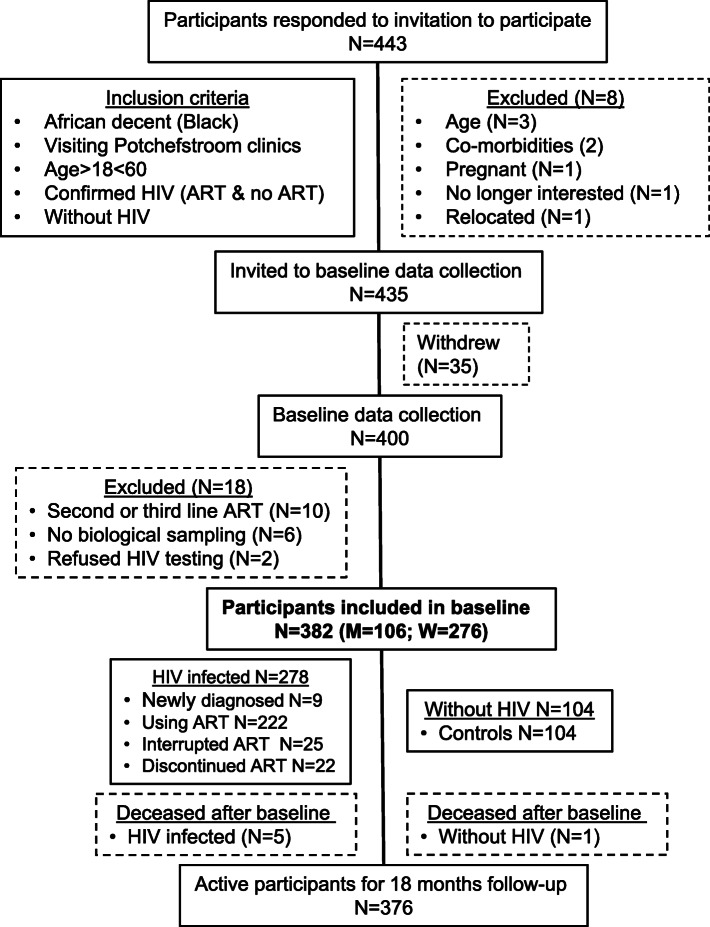
Diagram illustrating participation during the baseline data collection of the EndoAfrica-NWU study HIV: human immunodeficiency virus; ART: antiretroviral treatment.

Four hundred and forty-three volunteers (*n* = 443) showed interest in the study. Eight of the interested volunteers were excluded (Fig. 3) due to age, co-morbidities or being pregnant. Thus, 435 volunteers were invited to take part in the baseline data collection. Thirty-five of these volunteers did not attend the day of data collection and baseline data was collected from 400 volunteers.

Baseline data collection took place within the Hypertension Research and Training clinic on the Potchefstroom campus of the NWU from August 2017 to June 2018. Of the 400 volunteers, 382 (276 women and 106 men) met the eligibility criteria and were included in the study consecutively (Fig. 3). On the day of data collection participants with unknown HIV status and those who claimed to be HIV free were counselled and screened for HIV infection.

### Study participants

Three main study groups were planned for the EndoAfrica-NWU study based on the HIV and ART status of participants: (i) adults (18–60 years) with HIV, with no treatment or ≤ 4 weeks on first-line ART; (ii) adults with HIV on first-line ART for ≥4 weeks, and (iii) adults without HIV (controls). Of the 382 participants included in the baseline study, 278 were HIV infected and 104 were HIV free. Of the participants living with HIV, only nine participants were newly diagnosed as being HIV infected or used ART for less than 4 weeks as specified for group (i). The majority (*n* = 247) were on first-line ART (Table 1) on the day of data collection, while 22 discontinued their ART and were not using ART on the day of data collection. The first-line fixed dose combination ART consisted of 200 mg emtricitabine, a nucleoside reverse transcriptase inhibitor (NRTI), 300 mg tenofovir, also a NRTI, and 600 mg Efavirenze, a non-nucleoside reverse transcriptase inhibitor (NNRTI).

**Table 1:** Baseline profile of EndoAfrica-NWU participants

	HIV infected *n* = 278	HIV uninfected *n* = 104	*P-*value
Men, *n* (%)	74/278 (26.6)	32/104 (30.8)	0.42
Age (years)	42.7 ± 9.13	38.9 ± 11.7	0.004
Employed, *n* (%)	58/275 (21.1)	42/104 (40.4)	< 0.001
Education, *n* (%)			0.037
*None*	6/274 (2.19)	3/104 (2.89)	
*Primary*	62/274 (22.6)	24/104 (23.1)	
*Secondary*	187/274 (68.3)	60/104 (57.7)	
*Tertiary*	19/274 (6.93)	17/104 (16.4)	
**Anthropometry**			
Waist circumference (cm)	84.7 ± 13.5	87.2 ± 15.6	0.15
Body mass index (kg/m^2^)	26.2 ± 7.19	28.5 ± 8.24	0.013
**Cardiovascular profile**			
Hypertensive, *n* (%) ^a^	90/278 (32.4)	44/104 (42.3)	0.070
Systolic blood pressure (mmHg)	120 ± 21.6	123 ± 18.8	0.32
Diastolic blood pressure (mmHg)	84 ± 13.1	87 ± 14.8	0.052
Heart rate (beats/min)	72 ± 13.0	71 ± 14.0	0.50
Flow-mediated dilation (%) ^b^	7.44 (6.77;8.12)	7.23 (6.10;8.36)	0.75
Pulse wave velocity (m/s) ^b^	7.81 (7.66;7.97)	7.72 (7.46;7.98)	0.54
**Basic biochemical variables**			
Total cholesterol (mmol/L)	2.88 (1.71;4.42)	2.71 (1.80;4.09)	0.047
HDL (mmol/L)	1.00 (0.51;1.94)	0.89 (0.53;1.85)	0.010
LDL (mmol/L)	1.62 (0.78;2.86)	1.60 (0.86;3.07)	0.82
Triglycerides (mmol/L)	0.78 (0.35;2.16)	0.68 (0.30;1.93)	0.040
Glucose (mmol/L)	3.62 (2.75;5.11)	3.66 (2.67;7.61)	0.70
Glycated haemoglobin (%)	5.46 (4.80;6.53)	5.74 (4.90;10.5)	0.017
C-reactive protein (mg/L)	2.48 (0.25;16.1)	1.85 (0.13;20.1)	0.063
Gamma-glutamyltransferase (U/L)	51.2 (11.1;419)	22.3 (6.92;172)	< 0.001
eGFR (ml/min/1.73m^2^)	118 (92.5;147)	122 (97.9;151)	0.055
**Health behaviours**			
Tobacco use, *n* (%)	159/276 (57.6)	44/104 (42.3)	0.008
Alcohol use, *n* (%)	185/275 (67.3)	61/104 (58.7)	0.12
Physically active, *n* (%)	241/276 (87.3)	89/104 (85.6)	0.65
**HIV related variables**			
Duration of HIV > 5 years, *n* (%)	171/276 (62.0)	–	–
CD4 count (cells/μL)	417 (68.8;1121)	–	–
Viral load (copies/mL)	125 (10.0;93,433)	–	–
**Medication use**			
Antiretroviral therapy, *n* (%)			
*Never*	9/278 (3.23)	–	–
*Defaulted*	47/278 (16.9)	–	–
*Currently*	222/278 (79.9)	–	–
Duration of ART > 5 years, *n* (%)	124/265 (46.8)	–	–
Anti-hypertensive, *n* (%)	50/278 (18.0)	26/104 (25.0)	0.13
Statins, *n* (%)	6/278 (2.16)	6/104 (5.77)	0.072
Anti-diabetic, *n* (%)	2/278 (0.72)	5/104 (4.81)	0.008
Anti-inflammatory, *n* (%)	0/278 (0.00)	1/104 (0.96)	0.10

Data are expressed as arithmetic mean ± standard deviation, geometric mean (5th and 95th percentiles) or a percentage of *N*

*HIV* human immunodeficiency virus, *HDL* high-density lipoprotein cholesterol, *LDL* low-density lipoprotein cholesterol, *eGFR* estimated glomerular filtration rate, *ART* antiretroviral therapy

^a^Systolic blood pressure ≥ 140 mm Hg and/or diastolic blood pressure ≥ 90 mm Hg and/or use of antihypertensive medication

^b^Adjusted for systolic blood pressure and age, data expressed as mean (95%CI)

A policy of universal test-and-treat was introduced in South Africa on 1 September 2016, making ART available to all HIV infected individuals regardless of their cluster of differentiation 4 (CD4) T lymphocytes cell count. Same-day initiation, advocating ART initiation on the day of a patient’s HIV diagnosis, came into effect on 1 September 2017. As the EndoAfrica-NWU study commenced in mid-August 2017, these changes in the ART policy might have affected the recruited number of newly diagnosed patients or those that were on ART for < 4 weeks. Receiving an HIV diagnosis and needing to initiate ART may lead to internalized stigma (endorsing negative beliefs and feelings about PLHIV), which is particularly high in the first weeks after the diagnosis. Internalized stigma was associated with non-initiation of ART [18], and might also have played a role in the recruitment of these individuals for the study.

During recruitment for the study we found that some participants were defaulting (discontinuing or interrupting) their ART. Of those on ART the day of data collection (*n* = 247), 10% formerly interrupted their treatment and 22 participants (8,2% of those that were on ART in the past) were defaulting on the day of data collection. These participants reported to “feel better” and stopped taking their medication (ART). Defaulting ART may also be due to the mistaken belief that they have been healed when their viral load became undetectable [19]. Non-adherence to ART is the most common reason for treatment failure and may result in drug resistant HIV. In the State of the Epidemic Joint United Nations Programme on HIV/AIDS (UNAIDS) data report 2019, 67% of PLHIV in eastern and southern Africa were on treatment, but only 58% were virally suppressed [1]. Research identified the main determinants for non-adherence to be food insecurity, use of traditional herbs and medicine, stigma, issues with access to healthcare and disruption of services and medication distribution in sub-Saharan Africa [20, 21].

### Data collection procedures

**Fig. 4:**
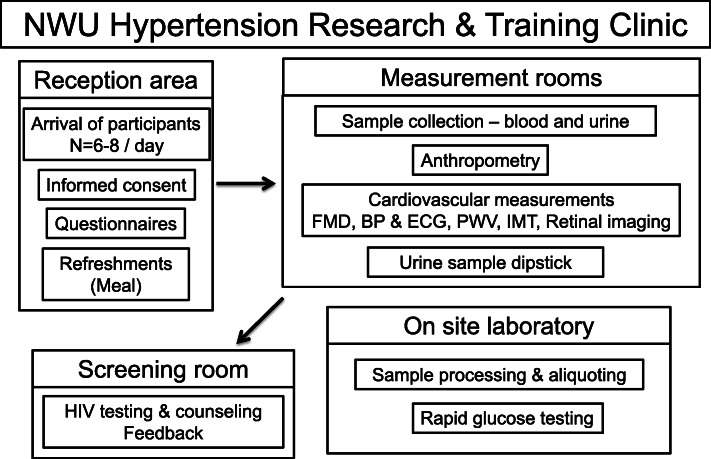
Flow diagram of the organizational procedures during data collection in the EndoAfrica-NWU study FMD: flow-mediated dilation; BP: blood pressure; ECG: electrocardiogram; PWV: pulse wave velocity; IMT: carotid intima-media thickness.

As participants showed interest in the study and met the inclusion criteria they were scheduled for data collection consecutively. Six to eight participants were scheduled per day and they were asked to fast from 22:00 the evening before data collection. The participants were transported free of charge to the clinic and were familiarized with the research environment of the clinic upon arrival at approximately 08:00. Data was collected under controlled conditions. After written informed consent was obtained, blood and early morning spot urine samples were collected (Fig. 4). All blood samples were immediately taken to the on-site research laboratory where blood glucose levels were determined with One Touch® Select (Johnson & Johnson, South Africa) test strips. The blood was centrifuged to obtain plasma or serum and aliquoted into cryovials. After the urine was tested with a Combur test strip (Roche Diagnostics, South Africa), it was also taken to the laboratory and aliquoted into cryovials. All samples were stored at − 80 °C until biochemical analyses could be performed.

In parallel, a questionnaire was completed, and anthropometric and cardiovascular measurements (Fig. 4) were performed. After data collection a meal was served. Individual feedback on the immediate test results and assessments were given by the registered research nurse and the participants were referred to a local clinic or doctor if required. The HIV status of the participants were determined with First response rapid HIV card test (Premier Medical Corporation Limited, Daman, India) and, if positive, confirmed with a Toyo rapid test (TurkLab, Turkey). The participants newly identified as HIV infected were referred to the clinic after counselling. After data collection (11:30–12:30 h), the participants were transported home. Blood samples of all the HIV infected participants were sent to the National Health Laboratory Service for viral load and CD4 cell count analyses. The results were taken to the participants during personal visits at their home address, in order for them to take it to their local clinic.

### Assessments

#### Questionnaire

A structured medical history of participants and their families (such as heart disease, stroke, hypertension, cancer and high cholesterol), lifestyle (smoking, alcohol consumption and eating habits) and medication use were obtained from the questionnaire.

#### Anthropometric measurements

Anthropometric measurements were performed according to standardized procedures with calibrated instruments and included body height (SECA 213 Stadiometer, SECA, Hamburg, Germany), body weight (SECA 813 Electronic scale, SECA, Hamburg, Germany), and waist and hip circumference (Lufkin steel anthropometric tape, W606PM; Lufkin, Apex, USA).

#### Flow-mediated dilation

Flow-mediated dilation (FMD) of the brachial artery is a non-invasive measurement technique and considered as a gold standard to measure macrovascular endothelial function [22]. Flow-mediated dilation is determined by the change in brachial artery diameter in response to a blood flow stimulus. This stimulus is created by releasing an arm cuff that was inflated to a supra-systolic blood pressure level. In response, nitric oxide (NO) is released from the endothelial cells and mediates the relaxation of the smooth muscle cells with subsequent widening of the artery [23].

The FMD procedure was performed by a trained observer, in a darkened room, while participants were in a supine position, and after they had rested for at least five minutes. After the procedure was explained, participants were asked whether they exercised, had anything to eat or drink, took any medications, or smoked since the previous night. A blood pressure cuff was fitted around the right forearm and the brachial artery was located on the right upper arm at around heart level. A longitudinal segment of the right brachial artery was visualized in B-mode with a 12 MHz linear probe, fixed in a single axis precision probe holder, and an Esaote MyLab™ Five ultrasound system (Esaote, Florence, Italy). The pulse repetition frequency was set at 6.7 kHz, after which depth was increased to 3 cm. After switching to pulse wave mode, the angle of insonation was set to + 60°. Flow velocity and diameter were continuously measured with edge-detection and wall tracking using Cardiovascular Suite™ Ultrasound Edition version 2.8.1 (Quipu srl, Italy) software. Baseline diameter was examined for one minute, inflation diameter for five minutes and the post-deflation diameter for three minutes. The software then automatically calculated baseline diameter, maximum diameter, recovery diameter, percentage FMD (the difference between baseline and maximum diameter, expressed as a percentage of baseline diameter), baseline shear rate and maximum shear rate.

#### Blood pressure and electrocardiogram

Duplicate brachial systolic blood pressure (SBP), diastolic blood pressure (DBP) and heart rate readings were obtained from the left arm of each participant using a validated OMRON M6 automatic digital blood pressure monitor (Omron Healthcare, Kyoto, Japan). Resting 12-lead ECG measurements were recorded in the supine position using the PC-ECG 1200 (Norvav Medical Ltd., Kiryat Bailik, Israel) apparatus.

#### Pulse wave velocity

The SphygmoCor® XCEL device (AtCor Medical, Sydney, Australia) was used to perform pulse wave analysis and determine carotid-femoral pulse PWV on the right-hand side with the participant in a supine position [24]. Pulse wave analysis was performed to produce an arterial wave form that provided brachial pulse pressure, estimated central SBP and central pulse pressure via a built-in generalized function [25]. Pulse wave velocity was measured using the femoral artery and carotid arterial waveforms which were captured simultaneously. To obtain these waveforms, an appropriate sized femoral cuff was placed on the upper right thigh and a tonometer was placed on the neck where the carotid pulse was felt the strongest. To determine the pulse wave travel distance, 80% of the distance measured between the two pulsating points (carotid-to-cuff measured using an infantometer, and femoral-to-cuff via a tape measure) was used [26]. Measurements were performed in duplicate, and repeated if PWV differed by more than 0.5 m/s.

#### Carotid wall analyses

Carotid intima-media thickness was measured non-invasively with the General Electric Vivid E9 (GE Vingmed Ultrasound A/S, Horten, Norway). A 3-lead ECG was attached with two electrodes just below the clavicles (left and right) and the third attached near the iliac crest on the participant’s left side. A transducer was used to obtain images and clips from two optimal angles, on both the left and right side of the participant. The images were then digitalized and imported into the carotid vessel analyser automated software (Vascular Research Tools 6, Medical imaging applications, Coralville, Iowa, USA). One investigator then determined carotid wall parameters, which included intima-media thickness and lumen diameter assessments. These parameters will be used in subsequent calculations to determine for instance cross-sectional wall area, carotid distensibility, Young’s elastic modulus and Beta-stiffness.

#### Retinal imaging

Prior to retinal imaging local anaesthetic drop (Novasine Wander 0.4% Novartis) was inserted into both eyes and intraocular pressure was measured using a Tono-Pen Avia applanation tonometer (Reichert 7–0908, ISO 9001, New York, USA). Retinal imaging was not performed on those with a high intraocular pressure or with a history of epilepsy. Retinal vessel imaging/assessments were done using the Dynamic Retinal Vessel analyser (DVA); fitted with a Zeiss Fundus Camera FF-450 Plus (Imedos, Jena, Germany). Images were taken 15–20 min after a drop containing 1% tropicamide and bensalconiumchloride 0.01% (m/v) (MYDRIACYL® 1% ophthalmic solution, *Alcon* Laboratories, South Africa) was administered to induce mydriatic conditions in the right eye. A colour retinal fundus image was captured by using Visualis 2.81 software (Imedos, Jena, Germany), with the camera angle set at 50°. The images were exported as Tagged Image File (TIF) format and retinal vessel calibers and pattern features such as vessel tortuosity, fractal analysis and lacunarity were determined using semi-automated MONO REVA (VITO, Belgium) software, as previously described [27].

In a subgroup of patients, dynamic retinal vessel responses to light flicker provocation were assessed. A 350 s protocol was used as described elsewhere [28]. The primary location for these assessments were the temporal superior retinal arterioles and venules located 0.5–2.0 optic nerve head diameters from the border of the optic nerve head. The raw numerical data generated from the measurement was exported to an Excel template (Microsoft), with macros. Parameters describing the vessel dilation, constriction and recovery phases were calculated as previously described [28].

#### Biological biomarkers

An array of biomarkers was analysed on site to evaluate cardiovascular risk and endothelial function. Plasma glucose levels were determined with the enzymatic reference method with hexokinase, and glycated haemoglobin (HbA1c) with a turbidimetric inhibition immunoassay from haemolysed whole blood. Components of the lipid profile, including total cholesterol (TC), high-density lipoprotein (HDL) cholesterol, low-density lipoprotein (LDL) cholesterol and triglyceride (TG) levels were determined in serum with enzymatic colorimetric methods. Serum apolipoproteins A-1 and B were determined with immunoturbidimetric assays. Liver enzymes, including alkaline phosphatase (ALP), alanine (ALT) and aspartate aminotransferase (AST) were determined with colorimetric methods according to the International Federation of Clinical Chemistry (without pyridoxal-5′-phosphate). Gamma-glutamyltransferase (GGT) was analysed with an enzymatic colorimetric assay in serum samples. C-reactive protein was determined with the high-sensitivity particle enhance turdidimetric assay, albumin with an immunoturbidimetric assay and creatinine with a kinetic colorimetric assay, based on the Jaffé method, in serum samples. All of the above mentioned, as well as urinary albumin, creatinine and ions were performed on the Cobas Integra® 400plus (Roche, Basel Switzerland).

Estimated glomerular filtration rate (eGFR) was calculated with the Chronic Kidney Disease Epidemiology (CKD-EPI) formula. Intercellular adhesion molecule-1, vascular cell adhesion protein-1, P-selectin, myeloperoxidase and growth differentiation factor-15 were determined with the Human Cardiovascular Disease Magnetic Bead panel 2 (HCVDP2, 96-Well Plate Assay, Luminex xMAP technology) kit on the Luminex 200 system (EMD Millipore®, Merck, Missouri, USA).

The CD4 cell count and viral loads of the HIV infected participants were analysed by the National Health Laboratory Services. The CD4 cell counts were determined using flow cytometric analysis (Beckman Coulter FC500 MPL/CellMek, Miami, FL). The Panleucogating (PLG) method was used for CD4 T-cell enumeration and the protocol uses a sequential automated gating strategy to identify all CD45+ leucocytes in order to measure the CD4% of lymphocyte and absolute CD4 count. The viral loads were assessed by using COBAS® AmpliPrep/COBAS® TaqMan® HIV-1 test, version 2.0. This test is a nucleic acid amplification test for the quantification of HIV 1 ribonucleic acid (RNA) in human plasma. The COBAS® AmpliPrep/COBAS® TaqMan® HIV-1 test, version 2.0 is based on three vital steps: (i) Isolation of HIV1 RNA from specimen; (ii) reverse transcription of RNA to complementary deoxyribonucleic acid (cDNA); and (iii) *Polymerase chain reaction* (PCR) amplification of cDNA and detection of cleaved dual labelled oligonucleotide detection probe specific to the target.

Although carotid wall and retinal imaging analyses were not available at the time of publication and do not form part the baseline profile results in this manuscript, the assessment methods are reported here as it forms part of the rational of the longitudinal EndoAfrica-NWU study.

### Follow-up data collection

A follow-up assessment was done 18 months after baseline data collection to determine progression or regression of the endpoints [17].

### Data management and handling

All the data generated were captured into the Research Electronic Data Capture (REDCap) system [29]. The baseline EndoAfrica-NWU study included 382 participants of whom 278 were HIV infected and 104 HIV free. In a sensitivity analysis, using G*power v3.1.9.2 software [30], we calculated that with our set group sizes, using an alpha error of 0.05 and power of 0.95, this study should be able to detect a medium effect size of 0.4.

We inspected continuous variables for normality using Q-Q plots, skewness and kurtosis. All variables with a non-Gaussian distribution were logarithmically transformed. Descriptive statistical analyses were used for inter-group comparisons (independent t-tests, analysis of covariance and Chi-square tests). We used IBM®, SPSS® version 26 (IBM Corporation, Armonk, New York) software for statistical analyses and the level for statistical significance was set at ≤0.05.

## Results

### Baseline profile

The baseline profile of the participants is indicated in Table 1. The majority of the participants were women, representing 73% of the HIV infected and 69% of the HIV free participants. The HIV infected participants (*n* = 278) were older (43 vs 39 years), less were employed (21% vs 40%), less had a tertiary education (9% vs 16%) and their body mass index (BMI) was lower (26 vs 29 kg/m^2^) than that of the HIV free participants (*n* = 104). More than half of the HIV infected participants (62%) were living with the virus for more than five years and 47% were on ART for more than five years.

The hypertension prevalence, SBP, DBP and heart rate did not differ between PLHIV and those free of HIV. Furthermore, after adjusting for SBP and age, FMD (7.44% vs 7.23%, *p* = 0.75) and PWV (7.81 m/s vs 7.72 m/s, *p* = 0.54) also did not differ between the participants with and without HIV. Basic biochemical analyses indicated higher TC, HDL, TG and GGT and lower HbA1c in the HIV infected group compared to the HIV free group (all *p* ≤ 0.047). The PLHIV reported higher tobacco use (*p* = 0.008) than those not infected with HIV.

## Discussion

The findings of the EndoAfrica-NWU study address the knowledge gaps with regards to HIV, endothelial function and cardiovascular risk markers in South Africa. Adults without HIV were included as a comparison group to determine whether HIV infection and the treatment thereof affects the cardiovascular disease risk markers and vascular function of PLHIV. We included traditional and novel biomarkers, as well as gold standard cardiovascular measurements.

Specific aspects of the baseline population profile of our population is noteworthy. More women than men were included as study participants, thereby a form of bias was introduced. However, of the total number of PLHIV older than 15 years in South Africa, an estimated 61% is women [1]. Epidemiological evidence also indicated that men in sub-Saharan Africa do not access HIV services as often as women [31], and are therefore also less inclined to partake in a HIV-related research study.

The average age of the study population was 42 years with PLHIV being somewhat older (43 vs 39 years). It is well known that the incidence and prevalence of CVD rise with age. This has also been reported previously in an HIV infected population where comorbidities seemed to develop earlier in PLHIV than in HIV free populations [32]. Despite that PLHIV in our study were older, and had higher TC and TG levels, their cardiovascular profile did not differ from those not infected. Even though the prevalence of hypertension also did not differ, elevated BP is well documented as a risk factor for the development of CVD, and we therefore adjusted for both age and SBP in the preliminary analyses of the endothelial and vascular function measurements. No difference in vascular function was observed, which was in line with another study where we indicated endothelial activation, but no increase in arterial stiffness in HIV infected compared to HIV free Africans [16]. In the latter study carotid dorsalis-pedis PWV was measured [16] and not the gold standard carotid-femoral PWV as in this study. Treatment did not seem to influence vascular function of the infected participants of whom 40% were on ART [16]. Nonetheless, further detailed analyses are needed to investigate the influence of HIV/ART on arterial stiffness in this study population with 79,9% of participants on ART. Whether the differences in cardiovascular risk markers, such as lipid- and glycaemic markers, GGT and tobacco use will affect the vascular measurements also remains to be seen.

The prevalence of HIV is indicated as 19% among adults 15–49 years old in South Africa, but the number is higher (22%) among women in this age range [2]. During recruitment for our study, young people did not volunteer and only 10% of the HIV infected participants in our study were younger than 30 years of age. This is in line with previous findings as historically, national AIDS programs struggled to persuade young people, who often underestimate their HIV risk, to periodically get tested for HIV [1]. Also, low numbers of young adults enter the ART program in South Africa, resulting in only a small percentage of young people being virally suppressed [33].

The increasing use of ART and the global obesity epidemic will likely accelerate the growing issues of weight gain, obesity, diabetes and CVD in PLHIV [34]. Although waist circumference did not differ between the HIV infected and HIV free participants in our study, the body mass index was lower (26 vs 29 kg/m^2^) among those infected (79.9% on ART). As in the case of the age differences, it remains to be determined whether adiposity will affect the cardiovascular outcomes of participants in this study.

A possible limitation of a study using FMD measurements to indicate endothelial function, is the low reproducibility of FMD measurements due to several influencing participant-related covariates and environmental factors [23]. However, studies have shown that the strict adherence to operating guidelines creates reliable FMD measurements for assessing endothelial function and cardiovascular risk [35–37]. We aimed for reproducible measurements and a low coefficient of variation for FMD by executing the standard operating procedures described here and in Strijdom et al. [17]. This included extensive training of the operators, regular quality control, and measuring the same brachial artery regions. We limited the number of FMD operators in the EndoAfrica-NWU study by using only two well trained researchers.

A further limitation of the study is that the recruitment of our participants was based on voluntary participation constituting a convenience sample. Despite efforts, we were unable to include more ART naïve, or individuals who were on ART for less than 4 weeks. In comparison to the high prevalence of HIV in South Africa, this is a relatively small study population. However, our study was performed under controlled conditions, approximately at the same time of day, and we used gold standard measurement techniques by a trained and experienced team of researchers in South Africa, the country with the biggest HIV epidemic in the world. Furthermore, our study reflects current health seeking behaviours of PLHIV in South Africa and will provide valuable insights which will contribute to the cardiovascular health of PLHIV in South Africa.

The baseline phase of the study has been completed and the 18-month follow-up data collection was completed in December 2019. The data analyses are ongoing, also on baseline carotid intima-media thickness and retinal imaging. The data is managed by a data manager using the RedCap system hosted at the Hypertension in Africa Research Team (HART), North West University, as well as the Department of Biomedical Sciences, Stellenbosch University (as part of the parent study).

## Conclusion

The EndoAfrica-NWU study addresses the lack of, and need for, research with regards to a detailed cardiovascular risk profile among South Africans living with HIV, in the epicentre of the global epidemic. While detailed analyses are still ongoing, the baseline profile of current data indicates no difference in the cardiovascular profile, including endothelial and vascular function measurements, despite the fact that the PLHIV were older. This study will generate knowledge unique to the South African HIV infected population and has the potential to lead to novel strategies to promote the cardiovascular health of this vulnerable population throughout Sub-Saharan Africa.

## Data Availability

The datasets used and/or analysed during the current study are available from the corresponding author on reasonable request. Potential collaborators are invited to apply to the corresponding author and to the principal investigator of the parent study [17] stating brief objectives of their project and analyses plan.

## Abbreviations

AIDS: Acquired immunodeficiency syndrome
ALP: Alkaline phosphatase
ALT: Alanine
ART: Antiretroviral treatment
AST: Aspartate aminotransferase
BP: Blood pressure
CD4: Cluster of differentiation 4
CKD-EPI: Chronic Kidney Disease Epidemiology
CRP: C-reactive protein
CVD: Cardiovascular disease
DBP: Diastolic blood pressure
DNA: Deoxyribonucleic acid
DVA: Dynamic vessel analysis
ECG: Electrocardiogram
eGFR: Estimated glomerular filtration rate
EndoAfrica-NWU: EndoAfrica-North-West University
FMD: Flow-mediated dilation
GDF-15: Growth differentiation factor-15
GGT: Y-glutamyltransferase
HART: Hypertension in Africa Research Team
Hb: Haemoglobin
HbA1c: Glycated haemoglobin
HDL: High-density lipoprotein
HIV: Human immunodeficiency virus
HREC: Health Research Ethics Committee
ICAM: Intercellular adhesion molecule
IMT: Intima-media thickness
LDL: Low-density lipoprotein
MPO: Myeloperoxidase
NNRTI: Non-nucleoside reverse transcriptase inhibitor
NO: Nitric oxide
NRTI: Nucleoside reverse transcriptase inhibitor
NWU: North-West University
PCR: Polymerase chain reaction
PLG: Panleucogating
PLHIV: People living with the Human Immunodeficiency Virus
PWV: Pulse wave velocity
REDCap: Research Electronic Data Capture
RNA: Ribonucleic acid
SBP: Systolic blood pressure
TC: Total cholesterol
TG: Triglyceride
UNAIDS: Joint United Nations Programme on HIV/AIDS
VCAM: Vascular cell adhesion molecule
